# A240 SOCIOECONOMIC STATUS AS A RISK FACTOR FOR ERCP OUTCOMES: A RETROSPECTIVE COHORT STUDY

**DOI:** 10.1093/jcag/gwae059.240

**Published:** 2025-02-10

**Authors:** M Mahjoob, K Khalaf, A Mokhtar, C Na, D Tham, N Sabrie, J Chon, M Meleka, S Abal, D Chopra, S Malipatil, S Gupta, S Jugnundan, W Mhalawi, N Gimpaya, J Mosko, C Teshima, G May, S Grover, N Causada Calo

**Affiliations:** Division of Gastroenterology, St. Michael’s Hospital, University of Toronto, Toronto, ON, Canada; Division of Gastroenterology, St. Michael’s Hospital, University of Toronto, Toronto, ON, Canada; Division of Gastroenterology, St. Michael’s Hospital, University of Toronto, Toronto, ON, Canada; Division of Gastroenterology, St. Michael’s Hospital, University of Toronto, Toronto, ON, Canada; Division of Gastroenterology, St. Michael’s Hospital, University of Toronto, Toronto, ON, Canada; Division of Gastroenterology, St. Michael’s Hospital, University of Toronto, Toronto, ON, Canada; Division of Gastroenterology, St. Michael’s Hospital, University of Toronto, Toronto, ON, Canada; Division of Gastroenterology, St. Michael’s Hospital, University of Toronto, Toronto, ON, Canada; Division of Gastroenterology, St. Michael’s Hospital, University of Toronto, Toronto, ON, Canada; Division of Gastroenterology, St. Michael’s Hospital, University of Toronto, Toronto, ON, Canada; Division of Gastroenterology, St. Michael’s Hospital, University of Toronto, Toronto, ON, Canada; Division of Gastroenterology, St. Michael’s Hospital, University of Toronto, Toronto, ON, Canada; Division of Gastroenterology, St. Michael’s Hospital, University of Toronto, Toronto, ON, Canada; Division of Gastroenterology, St. Michael’s Hospital, University of Toronto, Toronto, ON, Canada; Division of Gastroenterology, St. Michael’s Hospital, University of Toronto, Toronto, ON, Canada; Division of Gastroenterology, St. Michael’s Hospital, University of Toronto, Toronto, ON, Canada; Division of Gastroenterology, St. Michael’s Hospital, University of Toronto, Toronto, ON, Canada; Division of Gastroenterology, St. Michael’s Hospital, University of Toronto, Toronto, ON, Canada; Division of Gastroenterology, The Scarborough Hospital, University of Toronto, Toronto, ON, Canada; Division of Gastroenterology, St. Michael’s Hospital, University of Toronto, Toronto, ON, Canada

## Abstract

**Background:**

Endoscopic Retrograde Cholangiopancreatography (ERCP) has one of the highest adverse event rates among endoscopic procedures, close to 10%, including pancreatitis, bleeding, and perforation. To minimize complications and improve procedure success, understanding risk factors of adverse events is critical. Socioeconomic variables such as income, housing and education can influence health outcomes through access to resources, however, there is a literature gap regarding the effect of these variables on ERCP outcomes.

**Aims:**

The purpose of this study is to investigate socioeconomic predictors of ERCP outcomes.

**Methods:**

This study analyzed 6105 patients (51.4% female) who underwent ERCP at a tertiary care center between January 1, 2011 and December 31, 2020. Patient postal codes were translated to 2016 census division unique identifier codes which linked them to median monthly housing costs, household income, education levels, considered proxies for socioeconomic status. Multivariable logistic regression models were built to identify predictors of procedure success (achieving intended diagnostic or therapeutic goals) and adverse events (perforation, abdominal pain, pancreatitis, delayed/immediate bleeding). Data was analyzed using the “glm” package on R studio (Version 2023.06.1) and the adjusted P-value < 0.05 was considered for statistical significance.

**Results:**

The multivariable logistic regression model demonstrated that better access to housing (OR:1.11 [1.01, 1.12], p=0.02) is associated with procedure success. Conversely, older age (OR: 0.86 [0.78, 0.93], p<0.01) and male sex (OR=0.81 [0.69, 0.95], p<0.0001) are associated with lower odds of procedure success. Higher housing costs (OR: 0.86 [0.75, 0.97], p=0.04) are associated with decreased adverse events, after adjusting for age and sex.

**Conclusions:**

The findings of this study suggest socioeconomic differences may influence ERCP outcomes and should be further investigated in prospective studies.

**Funding Agencies:**

None

